# 3D Real-Time Echocardiography Combined with Mini Pressure Wire Generate Reliable Pressure-Volume Loops in Small Hearts

**DOI:** 10.1371/journal.pone.0165397

**Published:** 2016-10-24

**Authors:** Ulrike Herberg, Katharina Linden, Oliver Dewald, Eva Gatzweiler, Matthias Seehase, Georg Daniel Duerr, Jonas Dörner, Stephanie Kleppe, Dennis Ladage, Johannes Breuer

**Affiliations:** 1 Department of Pediatric Cardiology, Pediatric Heart Center, University of Bonn, Bonn, Germany; 2 Department of Cardiac Surgery, Pediatric Heart Center, University of Bonn, Bonn, Germany; 3 Department of Radiology, University of Bonn, Bonn, Germany; 4 Heart Center of the University Hospital Cologne, Dept. III for Internal Medicine, Cologne, Germany; University of Louisville, UNITED STATES

## Abstract

**Background:**

Pressure-volume loops (PVL) provide vital information regarding ventricular performance and pathophysiology in cardiac disease. Unfortunately, acquisition of PVL by conductance technology is not feasible in neonates and small children due to the available human catheter size and resulting invasiveness. The aim of the study was to validate the accuracy of PVL in small hearts using volume data obtained by real-time three-dimensional echocardiography (3DE) and simultaneously acquired pressure data.

**Methods:**

In 17 piglets (weight range: 3.6–8.0 kg) left ventricular PVL were generated by 3DE and simultaneous recordings of ventricular pressure using a mini pressure wire (PVL_3D_). PVL_3D_ were compared to conductance catheter measurements (PVL_Cond_) under various hemodynamic conditions (baseline, alpha-adrenergic stimulation with phenylephrine, beta-adrenoreceptor-blockage using esmolol). In order to validate the accuracy of 3D volumetric data, cardiac magnetic resonance imaging (CMR) was performed in another 8 piglets.

**Results:**

Correlation between CMR- and 3DE-derived volumes was good (enddiastolic volume: mean bias -0.03ml ±1.34ml). Computation of PVL_3D_ in small hearts was feasible and comparable to results obtained by conductance technology. Bland-Altman analysis showed a low bias between PVL_3D_ and PVL_Cond._ Systolic and diastolic parameters were closely associated (Intraclass-Correlation Coefficient for: systolic myocardial elastance 0.95, arterial elastance 0.93, diastolic relaxation constant tau 0.90, indexed end-diastolic volume 0.98). Hemodynamic changes under different conditions were well detected by both methods (ICC 0.82 to 0.98). Inter- and intra-observer coefficients of variation were below 5% for all parameters.

**Conclusions:**

PVL_3D_ generated from 3DE combined with mini pressure wire represent a novel, feasible and reliable method to assess different hemodynamic conditions of cardiac function in hearts comparable to neonate and infant size. This methodology may be integrated into clinical practice and cardiac catheterization programs and has the capability to contribute to clinical decision making even in small hearts.

## Introduction

Congenital heart diseases do have a prevalence of 107: 10,000 live births of which 29.4% are severe defects requiring early intervention [1]. Successful treatment and the resulting clinical outcome in children with congenital heart disease depend on cardiac size and function. The neonatal period until early infancy is the crucial time period for important and far-reaching decisions regarding operative, interventional and conservative therapy. In this age group, accurate assessment of ventricular volumes as well as systolic and diastolic performance is important for estimating disease progression and deciding on timing and choice of therapeutic interventions. Two-dimensional ultrasound plays an important role as a non-invasive diagnostic tool, but echocardiography alone does not provide sufficient evaluation of cardiac function, which is needed for the decision-making process [2]. Analysis of left ventricular pressure-volume loops (PVL) generates the afforded information about systolic and diastolic left ventricular function as well as ventricular-vascular coupling, and provides an important insight into cardiac physiology and the pathophysiology of congenital heart disease [3–6]. Conductance catheter technology (PVL_Cond_) is considered the gold standard for the determination of PVL and has been widely applied in basic and clinical research [3,4,7,8].

However, the acquisition of pressure-volume loops in small children is cumbersome and invasive due to the necessity of placing a large and stiff conductance catheter in the ventricle. Correct placement and control of position along the left ventricular axis which is essential for correct measurement requires repeated fluoroscopy and increased radiation exposure [9]. In most cases the smallest commercially available conductance catheter for human use (4F dependent on manufacturer requiring a 5F long sheath) is too large for neonates and small infants. Besides that it may not be possible to place such a large catheter in a neonate or infant, catheter related complications like arterial thrombosis increase with catheter size and the use of long sheaths [10–12]. Moreover, conductance technology requires repeated volume calibration to a reference method (e.g. thermodilution requiring more catheters) as well as determination of parallel conductance by repeated injection of concentrated saline using a second, separately placed catheter [9,13,14]. Hence, this method has not been introduced into daily pediatric practice or pediatric cardiac catheterization.

Throughout the cardiac cycle, cardiac volumes can be calculated nearly instantaneously, non-invasively and without the need for additional calibration, using real-time three-dimensional echocardiography (3DE) [15]. While systolic function could be evaluated using non-invasive blood pressure measurements only, the assessment of a complete cardiac cycle including diastolic function needs invasive pressure measurement. In a series of children and adolescents with congenital heart disease, we recently showed that calculation of PVL using 3DE in combination with simultaneous pressure measurements by a mini pressure wire (PVL_3D_) is feasible and reproducible [16].

The aim of this study was to validate the accuracy and reliability of left ventricular systolic and diastolic pressure-volume-relations under various hemodynamic conditions by comparing PVL_3D_ with the PVL_Cond_.

## Methods

### Animal protocol

This study was approved by the Institutional Animal Care and Use Committee of the State Office for Nature, Environment and Consumer Protection (Landesamt für Natur, Umwelt und Verbraucherschutz Nordrhein-Westfalen, Recklinghausen, Germany; 87–51.04.2010.A208). Animals were cared for in accordance to the principles of the European Convention for the Protection of Vertebrate Animals used for Experimental and Other Scientific Purposes. The whole study was performed under genereal anesthesia, and all efforts were made to minimize suffering.

Validation studies comparing PVL_3D_ with PVL_Cond_ were performed prospectively on 17 female piglets (landrace, mean body weight: 5.96 ± 1.26 [range 3.6–8.0] kg). In 8 additional female piglets (5.50 ± 0.53 [4.8–6.2] kg), the accuracy of 3D-volume calculations was compared to cardiac magnetic resonance imaging (CMR). Anesthesia, non-surgical and surgical procedures were identical in both animal groups.

### Experimental concept

In this preclinical prospective study left ventricular PVL_3D_ were compared to PVL_Cond_ under various hemodynamic conditions. Moreover, the accuracy of left ventricular, 3D-dervived volume calculations was assessed by direct comparison to volume calculations in CMR. All studies were analyzed by independent investigators in a blinded fashion.

#### Animal procedures

After intramuscular injection of ketamine (20 mg/kg), midazolam (0.5 mg/kg), and atropine (0.02 mg/kg), a peripheral venous catheter was placed and animals were anesthetized by continuous intravenous anesthesia with fentanyl (10 μg/kg/h), ketamine (5 mg/kg/h) and midazolam (0.2 mg/kg/h) throughout the experiment. Sufficient depth of anesthesia was ensured by monitoring heart rate and blood pressure for pain induced increases, testing of eyelid reflexes and reaction to cold and pain stimuli. If necessary, anesthesia was deepened by additional administration of fentanyl (10μg/kg) and/or ketamine (5 mg/kg). Muscle relaxation was performed throughout the procedure with pancuronium (0.2 mg/kg/h). Ventilation of the animals was done in a pressure-controlled mode (Evita 4 Dräger, Lübeck, Germany).

Prior to the insertion of the conductance catheter or the pressure wire, a 4F sheath (Terumo, Leuven, Belgium) was introduced into the right carotid artery. For preload reduction, a 10 mm dilatation balloon (Tyshak, NuMED Inc, Hopkinton, NY, USA) was inserted via a 5F sheath (Terumo, Leuven, Belgium), placed in a femoral vein. An additional central venous catheter (4F, trilumen, Arrow International, Reading, PA, USA) was placed into the left jugular vein. For transpulmonary thermodilution measurements (TPTD) a 4F 8 cm thermistor tipped femoral artery catheter (Pulsiocath; Pulsion Medical Systems, Munich, Germany) was placed in the right femoral artery as previously described [17]. Femoral arterial blood pressures, central venous pressures, expiratory CO_2_, transcutaneous saturations and ECG were monitored continuously. Arterial and venous blood gas analysis were performed before and after any change in hemodynamic conditions. At the end of the experiment the animals which were still in deep anesthesia were euthanized by intravenous injection of T61^®^ (Tetracainhydrochlorid, Mebezoniumiodid, Embutramid, 5ml).

### Assessment of pressure-volume relations

#### 3D-generated pressure-volume loops (PVL_3D_)

For the assessment of ventricular volume data, 3D datasets were obtained using a matrix transducer (Philips ie33, matrix-transducer X7-2, Andover, USA). For data acquisition and data analysis a standardized protocol was used [16,18]. Because of the retrosternal position of the porcine heart 3DE image quality is poor by a transthoracic (closed chest) approach. Therefore, 3DE baseline datasets were obtained both by a transthoracic approach and repeated by an epicardial (open chest) approach. 3D datasets of the left ventricle were acquired from the apical position during expiratory breath holding (full volume acquisition over 4–7 cardiac cycles). Depending on the depth of penetration, the mean 3D-volume frame rate ± SD was 36.5 ± 2.7 [range: 27–44] 3D volumes/s; corresponding to 14.7 ± 2.8 [11.1–27.9] 3D volumes per cardiac cycle respectively. End-diastolic volume (EDV), end-systolic volume (ESV), stroke volume (SV), and ejection fraction (EF) were calculated by using commercial semiautomatic software (QLab 8.1, Philips, Andover, USA). Here five points in two perpendicular planes, four mitral annular and one apical point, are placed manually on the left ventricle both in the enddiastolic and endsystolic frame. Then the semiautomatic software automatically tracks the endocardial boundaries. Manual correction of endocardial border delineation was performed if the boundaries were not detected correctly, especially in the apical and mitral annulus region [16].

Continuous pressure monitoring was performed after calibration of a 0.014 inch (0.36 mm) mini pressure wire (PressureWire^™^, Radi, St. Jude Medical, St. Paul, MN, USA) and its introduction into the left ventricle by a retrograde arterial approach without fluoroscopy. Data were recorded using the Radi Analyzer (Software Physiomon, St. Jude Medical, St. Paul, MN, USA) with a sampling rate of 200 Hz.

**Fig 1:**
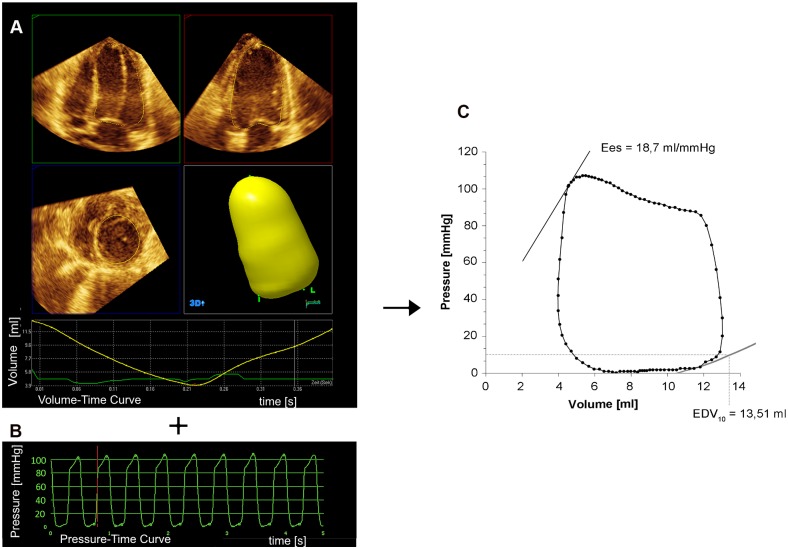
Principle of Pressure-Volume Relations derived from 3D real-time echocardiography and mini pressure wire. Volume-time curves calculated from 3DE-data (a) and pressure-time curves derived from mini-pressure wire (b) are synchronized and used to compute pressure-volume loops (c). Endsystolic elastance (Ees) and indexed enddiastolic volume at an enddiastolic pressure of 10 mmHg (EDV_10_) were estimated from a single-beat approach [19,20].

PVL were obtained by combination of the synchronized volume-time curves derived by 3DE and the simultaneously acquired pressure-time curves using customized software (Fig 1) [16].

Acquisition was performed three times under steady state in every hemodynamic condition examined. Systolic function was measured using end-systolic elastance (Ees), which was calculated by single-beat estimation method [19]. The effective arterial elastance (Ea) was calculated from the ratio of end-systolic pressure (ESP) to SV [10]. The Ees/Ea ratio was calculated to describe ventriculoarterial coupling [20].

For the assessment of diastolic function, the indexed EDV was calculated at an end-diastolic pressure of 10 mmHg (EDV_10_) as a practically load-independent parameter of ventricular chamber stiffness, using a single-beat approach [20]. The early active relaxation process (decay of the ventricular pressure during isovolumetric relaxation) is reflected by the isovolumetric relaxation constant tau. Tau was calculated using an exponential fit [21]. Maximal and minimal rate of pressure change over time (dp/dt_max_ and dp/dt_min_) were determined from continuous pressure measurement.

#### Pressure-volume loops using conductance technology (PVL_Cond_)

Immediately after obtaining PVL_3D_, a 3F micro-tip conductance catheter (SPR-923-3, Millar Instruments, Houston, TX, USA) was placed into the left ventricle using fluoroscopic guidance. After determination of parallel conductance by injection of 3 ml of 10% saline, PVL were recorded during expiratory breath hold and during preload reduction in three subsequent measurements and averaged**.** Determination of parallel conductance was done using Baan's equation for parallel conduction [13,14]. For volume calibration SV measured by 3DE using an average of three consecutive baseline measurements was used as reference [22,23].

ESP, end-diastolic pressure (EDP), dp/dt_max_, dp/dt_min_ and tau were calculated from the mean of at least 3 consecutive cardiac cycles. Linear fits estimated from recordings during preload reduction were used to calculate load-independent end-systolic pressure-volume-relations (Ees). Correspondent to assessment of diastolic function with 3DE and pressure wire EDV10 was calculated at an end-diastolic pressure of 10 mmHg.

#### Experimental protocol with variation of hemodynamic conditions

**Fig 2:**
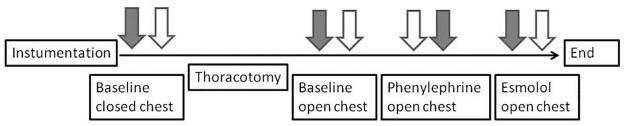
Protocol of the experiment. Solid arrows represent acquisition of PVL_3D,_ Unshaded arrows represent acquisition of PVL_Cond._

Baseline measurements of PVL_3D_ and PVL_Cond_ were performed under closed and open chest conditions. As poor 3DE image quality on a closed chest results in inappropriate volume calculations [24], distal partial sternotomy was performed to expose an appropriate apical ventricular area after baseline measurements had been taken (Fig 2). Once the baseline measurements had been repeated on the open chest, hemodynamic conditions were modified by increasing the afterload (alpha-adrenergic stimulation with phenylephrine: bolus 10 μg/kg followed by 10 μg/kg/min continuous infusion (Baxter, Deerfield, Il, USA)). After a washout period of 20 minutes, beta-adrenoceptor blockage was started (esmolol 1mg/kg repeatedly (Baxter, Unterschleißheim, Germany)) using the decrease of dp/dt_max_ and P_max_ of more than 20% as indicators for effectiveness of esmolol dose [24,25]. During each hemodynamic state PVL_3D_ and PVL_Cond_ were obtained successively. For 3DE acquisition the conductance catheter was removed to avoid artifacts that could impair left ventricular volume calculations.

### Cardiac magnetic resonance imaging

3DE was performed on closed chests just before the CMR and was repeated on closed and open chests after CMR.

CMR was performed on a 3 Tesla whole body scanner (Ingenia, Philips Healthcare, Best, Netherlands). Retrospectively gated balanced steady state free precision (SSFP) cine images [26] with 30 heart phases, median 27 frame/second (23–35 frames/second) were acquired for the standard heart axes using a breath holding technique to compensate for motion. Scan parameters for SA SSFP cine images were: field-of-view 320 mm; time to echo time/repetition time 1.6/3.2 ms; slice thickness 4 mm; flip angle 45°; and in-plane resolution 1.5 mm.

For each slice and heart phase, endocardial borders (including papillary muscles) were traced manually in a short axis data set, covering the whole left ventricle using a dedicated analysis software (View Forum, Philips, Best, Netherlands). Left ventricular EDV, ESV, EF and SV were calculated.

### Statistical Analysis

All data were analyzed using Graph Pad Prism 6 (GraphPad Software, San Diego, CA, USA) and SPSS version 22 (SPSS, inc, Chicago, Il, USA). For Bland-Altman-analyses for multiple observations per individual MedCalc for Windows, version 12.5 (MedCalc Software, Ostend, Belgium) was used. We used a subset of 10 randomly selected animals to determine intra- and inter-observer variability of 3DE-derived data by two different observers who were blinded to the previous measurements. Time between intraobserver measurements was 4 weeks. Intra- and inter-observer variability was expressed by the coefficient of variation (standard deviation (SD)/mean), and Bland-Altman analysis.

Agreement of PVL_Cond_ and PVL_3D_ as well as open and closed chest measurements were compared by Bland-Altman analysis or by Bland-Altman analysis for multiple observations per individual [27]. Correlation was assessed by calculation of the Intra-Class Correlation Coefficient (ICC). The differences between pharmacologic conditions were analyzed using paired Student t-tests or Wilcoxon signed-rank tests, as appropriate. Differences were considered to be significant when the p-value <0.05.

CMR and 3DE volume calculations were compared using Bland-Altman analysis.

## Results

### PVL—comparison of 3DE and Conductance-Technology

**Fig 3:**
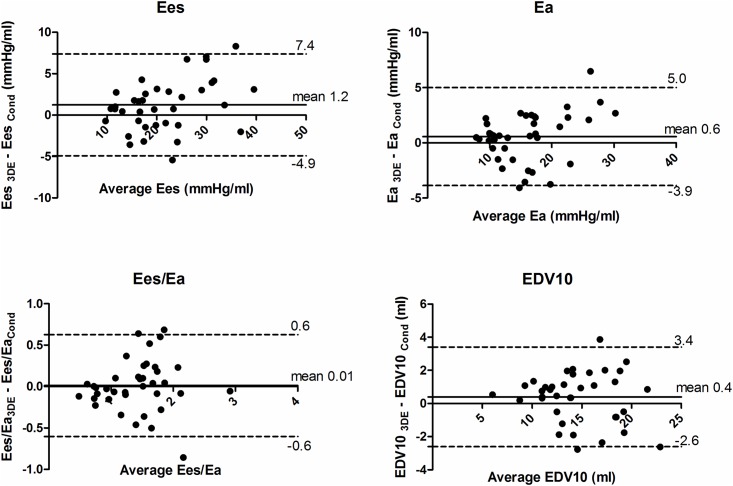
Bland–Altman plot for multiple observations per individual for parameters obtained from PVL_3D_ and PVL_Cond._ Solid line represents mean bias. Dashed lines represent limits of agreement. Ees, endsystolic elastance; Ea, arterial elastance; Ees/Ea, ventriculoarterial coupling; EDV_10_, indexed enddiastolic volume at an enddiastolic pressure of 10 mmHg.

Overall, there was a good agreement between both methods, and end-systolic and end-diastolic pressure-volume-relations derived from PVL_3D_ correlated well with those calculated from PVL_Cond_. Bland-Altman plots were calculated for each hemodynamic condition (Table 1 and S1 Table). Bland-Altman plots for multiple observations per individual are shown in Fig 3. Table 2 shows the correlation of both methods.

**Table 1:** Bland-Altman analysis for parameters obtained from PVL_3D_ and PVL_Cond_ under different hemodynamic conditions.

	Baseline		Phenylephrine		Esmolol	
	Bias ± SD	LOA	Bias ± SD	LOA	Bias ± SD	LOA
Ees [mmHg/ml]	0.04 ± 2.6	-5.07–5.14	2.09 ± 4.19	-6.12–10.31	1.65 ± 1.81	-1.9–5.19
Ea [mmHg/ml]	-0.21 ± 1.8	-3.73–3.31	1.61 ± 1.71	-1.73–4.96	0.39 ± 2.85	-5.2–5.98
Ees/Ea	0.01 ± 0.43	-0.83–0.84	0.0 ± 0.22	-0.42–0.43	0.03 ± 0.27	-0.49–0.56
EDV_10_ [ml]	0.72 ± 1.08	-1.4–2.85	0.04 ± 1.63	-3.15–3.23	0.44 ± 1.89	-3.27–4.15
ESP [mmHg]	2.15 ± 4.57	-6.8–11.12	2.9 ± 7.6	-12–18	6.4 ± 9.2	-11.63–24.35
tau [s]	0.69 ± 2.11	-3.43–4.82	0.5 ± 2.56	-4.53–5.52	0.53 ± 2.6	-4.56–5.62

SD, standard deviation; LOA, limits of agreement; Ees, endsystolic elastance; Ea, arterial elastance; Ees/Ea, ventriculoarterial coupling; EDV_10_, indexed enddiastolic volume at an enddiastolic pressure of 10 mmHg; ESP, endsystolic pressure.

**Table 2:** Correlation of pressure-volume loop parameters obtained by 3DE (PVL_3D_) and conductance technology (PVL_cond_) under different hemodynamic conditions (Intraclass-Correlation Coefficient ICC with 95% CI).

**Baseline**				
	**PVR_3D_** mean ± SD	**PVR_Cond_** mean ± SD	**ICC**	**95% CI**
ESP [mmHg]	94.86 ± 15.21	92.09 ± 16.48	0.977	0.926–0.992
Ees [mmHg/ml]	19.18 ± 6.0	19.14 ± 5.63	0.951	0.838–0.985
Ea [mmHg/ml]	11.36 ± 2.52	11.57 ± 4.01	0.927	0.762–0.978
Ees/Ea	1.63 ± 0.38	1.62 ± 0.41	0.819	0.307–0.953
tau [s]	19.25 ± 3.46	18.56 ± 3.57	0.899	0.682–0.969
EDV_10_ [ml]	14.34 ± 4.21	13.62 ± 4.01	0.976	0.893–0.993
Heart rate [/min]	124 ± 22	125 ± 22	0.875	0.639–0.957
**Phenylephrine**				
	**PVR**_**3D**_	**PVR**_**Cond**_	**ICC**	**95% CI**
ESP [mmHg]	141.3 ± 25.86	136.7 ± 27.78	0.949	0.844–0.984
Ees [mmHg/ml]	28.25 ± 9.59	26.16 ± 7.63	0.928	0.756–0.978
Ea [mmHg/ml]	20.73 ± 6.21	19.11 ± 5.63	0.962	0.307–0.952
Ees/Ea	1.46 ± 0.68	1.45 ± 0.64	0.975	0.916–0.992
tau [s]	24.45 ± 9.18	23.95 ± 9.28	0.981	0.940–0.994
EDV_10_ [ml]	14.68 ± 3.0	14.64 ± 3.24	0.932	0.775–0.979
Heart rate [/min]	129 ± 25	123 ± 24	0.972	0.819–0.992
**Esmolol**				
	**PVR**_**3D**_	**PVR**_**Cond**_	**ICC**	**95% CI**
ESP [mmHg]	104.2± 19.92	97.3 ± 13.82	0.977	0.926–0.992
Ees [mmHg/ml]	18.44 ± 6.62	16.79 ± 5.92	0.951	0.838–0.985
Ea [mmHg/ml]	16.85 ± 5.67	16.36 ± 3.99	0.927	0.762–0.978
Ees/Ea	1.11 ± 0.43	1.01 ± 0.32	0.819	0.307–0.952
tau [s]	26.67 ± 9.23	26.16 ± 9.86	0.899	0.682–0.969
EDV_10_ [ml]	15.37 ± 4.27	14.93 ± 4.65	0.976	0.893–0.993
Heart rate [/min]	128 ± 17	124 ± 17	0.875	0.639–0.957

SD, standard deviation; CI; confidence interval; ESP, endsystolic pressure; Ees, endsystolic elastance; Ea, arterial elastance; Ees/Ea, ventriculoarterial coupling; EDV_10_, indexed enddiastolic volume at an enddiastolic pressure of 10 mmHg; ICC, intraclass correlation coefficient

#### Response to pharmacologic agents

**Fig 4:**
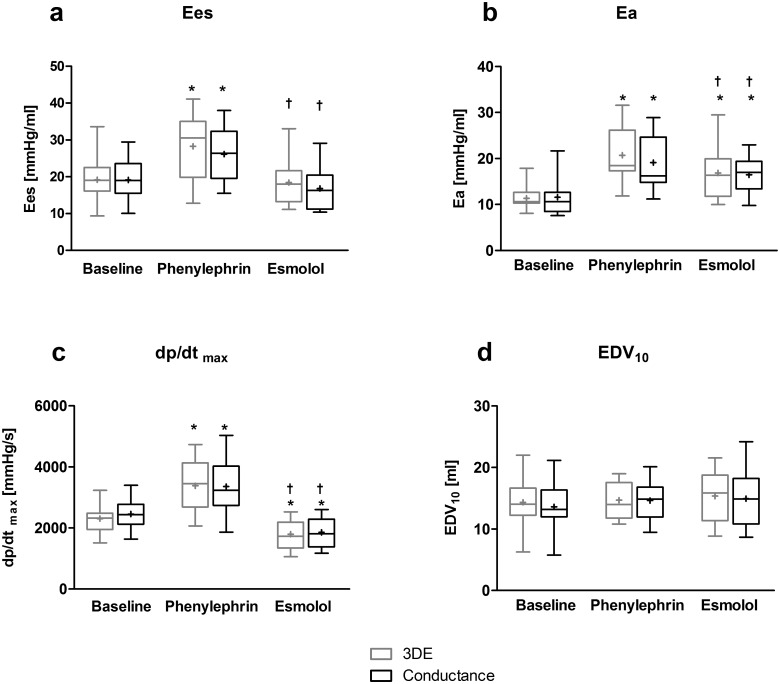
Comparison of PVL_3D_ (gray box) and PVL_Cond_ (black box) under various hemodynamic conditions. Significant changes of (a) endsystolic elastance (Ees), (b) arterial elastance (Ea), (c) peak positive rate of pressure over time (dp/dt_max_), as well as (d) indexed enddiastolic volume (EDV_10_) induced by phenylephrine and esmolol were consistently detected by both methods. (n = 17 piglets), paired Student t-tests. * = significant compared to baseline (p<0.05), † = significant compared to phenylephrine (p<0.05), + = mean, box = 25^th^ to 75^th^ percentile, the bar in the box indicates the median, whiskers indicate the range.

**Fig 5:**
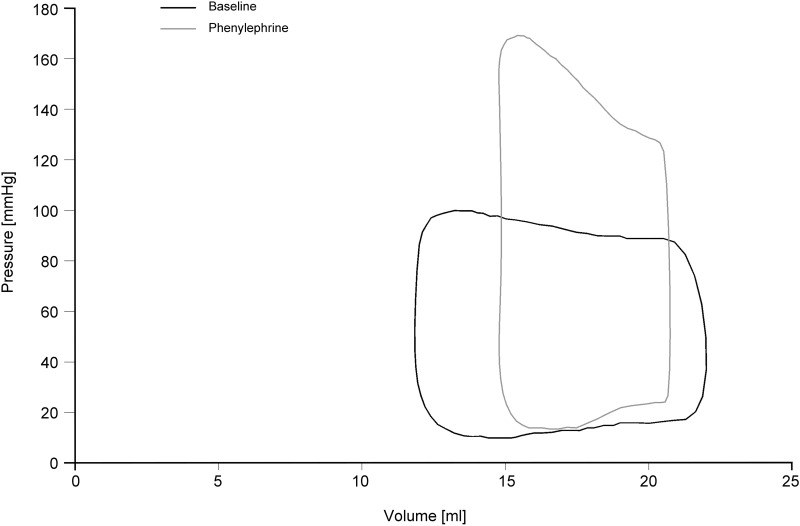
Example of Pressure-Volume Loops derived from 3D real-time echocardiography and mini pressure wire under various hemodynamic conditions. Pressure volume loops at baseline (black line) and phenylephrine (grey line) depicting the decrease of SV and the increase of Ees, Ea, and P_max_ during alpha-adrenergic stimulation with phenylephrine. Baseline: P_max_ = 100 mmHg; SV = 9.9 ml; Ees = 18.3 mmHg/ml; Ea = 9.8 mmHg/ml; Ees/Ea = 1.87; dp/dt_max_ = 2293 mmHg/s; dp/dt_min_ = -2163 mmHg/s; EDV_10_ = 19.7 ml; Tau = 18 s. Phenylephrine: P_max_ = 169 mmHg; SV = 6.0 ml; Ees = 43.2 mmHg/ml; Ea = 28 mmHg/ml; Ees/Ea = 1.54; dp/dt_max_ = 3641 mmHg/s; dp/dt_min_ = -2739 mmHg/s; EDV_10_ = 17.6 ml; Tau = 26.5 s.

Both methods reflected the same changes induced by the pharmacologic agents (Fig 4). During phenylephrine and esmolol application, heart rate and EDV did not change significantly, whereas SV and EF decreased when compared to baseline (S2 Table). Stimulation with phenylephrine affected mainly systolic function with significant increases in Ees, Ea, P_max_ and dp/dt_max_ in both conductance and 3DE measurements (Fig 4 and representative example in Fig 5). Diastolic function (EDV_10_, tau, dp/dt_min_) did not change significantly during phenylephrine administration. The esmolol-induced changes were comparable between PVL_3D_ and PVL_cond_. Application of esmolol resulted in a decrease in ESP, dp/dt_max_, Ees and EF.

#### Intra- und inter-observer variability

Variation coefficients of intra- and inter-observer variability in parameters calculated from PVL_3D_ were less than 5% (Table 3).

**Table 3:** Intra- and inter-observer variability of parameters generated from PVL_3D_.

	Coefficient of variation	Bland-Altman analysis	
	Mean ± SD in %	Bias +/- SD in %	LOA in %
**Intra-observer**			
Ees	2.79 ± 3.53	0.06 ± 6.5	-12.80–12.67
Ees/Ea	3.07 ± 3.60	-2.33 ± 4.24	-10.63–5.97
Tau	0.07 ± 0.16	0.09 ± 0.06	-0.48–0.66
EDV_10_	2.55 ± 2.18	-2.33 ± 4.24	-10.63–5.97
**Inter-observer**			
Ees	2.61 ± 3.34	1.19 ± 5.92	-10.42–12.8
Ees/Ea	2.83 ± 3.49	- 0.31 ± 2.08	-4.18–3.96
Tau	0.75 ± 1.42	-0.29 ± 2.25	-4.12–4.69
EDV_10_	1.2 ± 0.79	0.69 ± 6.4	-11.85–13.23

SD, standard deviation; LOA, limits of agreement; Ees, endsystolic elastance; Ea, arterial elastance; EDV_10_, indexed enddiastolic volume at a pressure of 10 mmHg

### Comparison of volume calculations between 3DE and CMR

For volume measurements there was a good agreement between CMR and 3DE (Table 4). SV assessed by TPTD were significantly higher than by CMR (mean bias 2.3 ± 2.2 ml, limits of agreement -2.1–6.7 ml). They showed less agreement to CMR than 3DE. In the same animals in which CMR was performed and compared to 3DE we compared 3DE with TPTD and found mean bias of -2.3 ± 2.2 ml with limits of agreement of -7.3–2.7 ml.

**Table 4:** Bland-Altman analysis for volumetric parameters obtained by 3DE and CMR.

	Agreement Bland-Altman—CMR-3D	
	Bias ± SD	LOA
EDV [ml]	-0.03 ± 1.35	-2.66–2.61
ESV [ml]	0.10 ± 0.76	-1.38–1.58
SV [ml]	-0.13 ± 1.2	-2.43 ± 2.18
EF [%]	-0.5 ± 5.8	-11.8–10.8

SD, standard deviation; LOA, limits of agreement; EDV, enddiastolic volume; ESV, endsystolic volume; SV, stroke volume; EF, ejection fraction

#### Closed vs. open chest measurements

We observed higher ventricular volume in open- than in closed-chest 3DE measurements (bias±SD: EDV 2.0±2.8 ml; SV: 0.4±1.5 ml; ESV 1.6± 2.3ml, P_max_ 0.7 ± 8.4 mmHg, tau 0.5 ± 5.5 ms (S3 Table).

## Discussion

This study proved that 3DE-derived pressure-volume relations are comparable to those obtained by the current gold standard conductance catheter technique. In small hearts equivalent to neonatal and infant cardiac size, both methods showed good agreement under various pharmacological conditions.

Previously, in a pilot study, we demonstrated the feasibility of PVL_3D_ in children of different ages suffering from a variety of heart disease [16]. In the present study, we were able to confirm our findings in a preclinical model of small hearts and show that PVL_3D,_ calculated from single-beat estimations, correlated well with those PVL_Cond_, obtained during preload reduction.

### Validation of volumetric data

PVL are dependent on the accurate assessment of volume as well as pressure changes. As a basic requirement, we validated the accuracy of 3DE volume measurements in piglet hearts of neonatal and small infant size. We demonstrated that determination of left ventricular volumes by 3DE is accurate when compared to CMR. Our findings are in accordance with the previous study of Friedberg et al. in small children with congenital heart disease [28].

### Validation of pressure-volume loops

PVL_3D_ correlated well in respect to the major parameters of systolic as well as diastolic function when compared to PVL_Cond_. It is important to emphasize that PVL_3D_ and PVL_Cond_ use different algorithms for the acquirement of the loops and calculation of end-systolic and end-diastolic pressure-volume relations. PVL_Cond_ estimates volume changes based on changes of blood conductivity and uses transient preload reduction over multiple beats for the calculation of Ees. In contrast, PVL_3D_ rely on a single pressure-volume loop, affording single-beat estimations [19,20]. Dp/dt_max_, dp/dt_min_, P_max_, EDP and tau are parameters that originate solely from pressure-time curves. In contrast, ESP, EDV_10_, Ees and Ea are derived from pressure-volume loops. However, we found that not only the pressure-dependent parameters, but also Ees, EDV_10_ and Ea were well correlated. Slightly wider limits of agreements and ICC 95% CI can be due to the necessity of removing one catheter before the measurement with another catheter. The two methods showed concordant changes of systolic and diastolic pressure-volume-relations both under baseline and altered hemodynamic conditions.

### Detection of hemodynamic changes

Alpha-adrenergic stimulation by administration of phenylephrine notably affected systolic function while diastolic parameters did not change significantly. Phenylephrine, an alpha-1 agonist, causes peripheral vasoconstriction and thus increases afterload, which was directly reflected by a significant increase in Ea [29]. Consequently, SV and EF decreased, whereas EDV and EDV_10_ remained essentially unchanged. Using higher doses of phenylephrine (10 μg/kg/min) than Cassidy et al. (2 μg/kg/min) [30], we observed a significant increase in Ees. The few studies that have evaluated ventricular performance under the influence of esmolol in piglets showed a significant decrease in Ees [24,25] and our results confirm these data. Application of esmolol after adrenergic stimulation resulted in a decrease in ESP, dp/dt_max_, dp/dt_min_, Ees and EF as well as an increase in ESV, EDV and EDP [25].

### Technical aspects and considerations

To achieve satisfactory temporal and spatial resolution in the fast-beating infant heart, 3D full-volume datasets have to be used and recorded over 4–7 cardiac cycles. In consequence, beat-to-beat variability cannot be assessed by 3D full-volume acquisition mode. The echocardiographic evaluation of beat-to-beat variability is only possible using the technique of single-beat acquisition of 3D datasets with low temporal and spatial resolution [31]. Kutty et al. [22] integrated 3D single-beat volume data derived by transesophageal 3DE (TEE) with left ventricular pressure curves from a conductance catheter in adult pig hearts. Despite using TEE, their temporal resolution was poor compared to our study (12–14 3D volumes/s vs. 36.5 ± 2.7 3D volumes/s). Moreover, only the systolic parameter, Ees, and no diastolic parameters were calculated in contrast to our data. We have to emphasize, that the 3D single-beat acquisition mode cannot provide sufficient temporal and spatial resolution for accurate volume calculation in the fast-beating, small pediatric heart.

Besides conductance catheter technique, several alternative methods have been developed to assess PVL. CMR-derived PVL have been tested in pigs and humans [6,32]. In small children, this method is unfavorable, because CMR is performed under general anesthesia and pressure is recorded simultaneously with less-accurate, fluid-filled catheters compared to high-fidelity micromanometers. CMR volumetry also requires data acquisition over several heartbeats and does not assess beat-to-beat variability. Moreover, CMR is time-consuming and expensive so that this method has not been routinely applied and is, at least currently, limited to research purposes.

Fully non-invasive approaches based on 3DE and radial tonometry [33] are promising, but unfortunately are limited to recording only systolic parameters (Ees, Ea) and neglect diastolic function. For the assessment of diastolic PVL, the determination of pressure data from a complete cardiac cycle is indispensable. In the light of these facts, our results provide a solid ground for clinical application especially during planned and necessary cardiac catheterization of PVL_3D_ in pediatric hearts.

### Limitations

For 3D volume measurement, an adequate imaging window is essential. Underestimation of 3D volume calculations can be related to blurred imaging of ventricular borders and difficult contour detection on post-processing. We demonstrated in our pilot study [16] that left ventricular PVL can easily be generated by transthoracic echocardiography in children. Moreover our group examined 633 left ventricles by 3DE in healthy children aged 0–18 years (median 10 years). We were able to obtain measurable data for the left ventricle in 92% of all healthy children and in 87% of children aged 0–36 month (unpublished data). Renella et al. found in his study that it is feasible to obtain measurable right ventricular 3DE datasets in 80% of patients aged 0–3 years [34]. Because of a retrosternal position of the porcine heart we observed poor 3DE image quality in a closed chest model. Therefore, we decided to perform thoracotomy to improve the imaging window and thus volume measurements. However, hemodynamic data were comparable between open and closed chest conditions and were not influenced by thoracotomy.

For technical reasons, PVL_3D_ and PVL_Cond_ could not be obtained simultaneously. In contrast to findings in adult pigs [22], we observed intracavital echocardiographic artifacts in the small ventricles of piglets produced by the conductance catheter which impaired the semiautomatic contour analysis of left ventricular borders during 3DE volume calculation. Therefore the conductance catheter had to be removed during 3DE image acquisition. On the other hand, the mini pressure wire disturbed conductance measurements and could not be left in place during conductance analysis. The removal and repositioning of the high-fidelity pressure catheters may have contributed to small hemodynamic changes between the consecutive measurement of PVL_3D_ and PVL_Cond_ and might have resulted in wider limits of agreement and confidence intervals. As stated in the work of Hapfelmeier et al. hemodynamics such as cardiac output change from assessment to assessment. So a scenario with real repetition of measurements is hardly possible. In these cases Bland-Altman plots are still the best way to interpret the data [35].

For volume measurements, the conductance catheter has to be calibrated with a reference method. Often this reference method is a thermodilution method. We also measured SV with TPTD and found a high bias with wide limits of agreement compared to CMR. We also compared 3D-derived volume measurements with CMR data and found that 3DE correlated better with CMR data than TPTD measurements and showed a smaller bias and limits of agreement. Therefore we decided to calibrate the conductance catheter with volumes obtained by 3DE which were closer to the CMR data. Moreover, we sought to examine the agreement of PVL_3D_ and PVL_Cond_ regarding detection and tracing of hemodynamic changes. Basing the calibration of volumetric data on the same reference in the beginning enabled us to study the tracking of hemodynamic changes of the two methods without an additional bias of a third volumetric method like TPTD. We cannot rule out that this might have led to bias.

### Clinical significance

Accurate measurement of cardiac size and function is particularly important in neonates and infants because fundamental decisions concerning conservative, interventional or operative therapy have to be made. In patients with borderline ventricles (uni- vs. biventricular palliation) [36], or single ventricle physiology [37], therapeutic options and long term outcome rely on the prediction of systolic as well as diastolic ventricular function. Most accurate evaluation of functional parameters in addition to morphometric indices will aid determination of the optimal therapeutic approach [38]. In older children, precise assessment of cardiac function is vitally important with chronic pressure- and volume-loaded ventricles and pediatric heart failure [5,39]. In addition, pharmacologic effects on ventricular function can be evaluated and the therapy adjusted to the patient’s hemodynamic condition. Moreover, PVL_3D_ could easily be applied in adults, e.g. during TEE-guided transcatheter aortic valve implantation or mitral valve clip procedure [23].

The calculation of ventricular volumes measured by conductance technology is based on Ohm´s law, based on the assumption that the ventricle can be regarded as several stacked cylindrical segments. However, ventricular shape in congenital heart disease does not always meet this ideal geometric assumption. Central positioning of the catheter along the ventricular axis is also required for exact measurement of volume changes. Therefore abnormal shape of the ventricle or incorrect placement of the catheter influences the measurements by conductance technique. 3DE has the potential to correctly assess volumes even in malformed ventricles [28]. Studies comparing PVL_3D_ and PVL_Cond_ are lacking so far. Therefore we decided to first do a validation for PVL_3D_ compared to the goldstandard conductance technique in normally shaped small hearts to avoid further bias by these mentioned limitations of the conductance technique.

Compared to the conductance technology, PVL_3D_ requires smaller vascular access and does not need additional fluoroscopy. Insertion of the pressure wire and measurement is easier and faster than that of the conductance catheter whose correct placement along the axis of the ventricle can be cumbersome, time-consuming and requires repeated fluoroscopy. Therefore PVL_3D_ can be included into routine catheterization programs with minimal additional effort and has the potential to provide correct PVL even in malformed hearts as often found in congenital heart defects. In future, totally noninvasive approaches based on noninvasive pressure measurements and 3D-derived volume-time curves may improve serial assessment of cardiac function in daily practice.

### Conclusion and perspective

We have demonstrated that the combination of 3DE and mini pressure wire measurements provides a reliable assessment of PVL compared to conductance catheter technique. This novel method is sensitive and accurate in detecting hemodynamic changes in the juvenile, mammalian heart. Due to its severe limitations, conductance technology is unlikely to be used as routine procedure to assess ventricular function. Our 3DE-derived PVL measurement protocol is a promising new approach to determine cardiac function in clinical settings especially during cardiac catheterization programs.

## Supporting Information

S1 Table Bland-Altman analysis for further parameters obtained by 3DE and conductance technology under different hemodynamic conditions. (DOCX)

S2 Table Comparison of parameters obtained by 3DE and conductance technology under different hemodynamic conditions (Student t-test; mean ± SD). (DOC)

S3 Table Comparison of parameters obtained by 3DE between open vs. closed chest measurements at baseline conditions. (DOCX)
