# Supplementary material for: 3D Real-Time Echocardiography Combined with Mini Pressure Wire Generate Reliable Pressure-Volume Loops in Small Hearts
Source: PLoS One. 2016 Oct 24;11(10):e0165397. doi: 10.1371/journal.pone.0165397 (PMC5077139; doi:10.1371/journal.pone.0165397)
Supplement: S1 Table — (DOCX) [file pone.0165397.s001.docx]

## S1 Table. Bland-Altman analysis for further parameters obtained by 3DE and conductance technology under different hemodynamic conditions.

|  | **Baseline** | | **Phenylephrine** | | **Esmolol** | |
| --- | --- | --- | --- | --- | --- | --- |
|  | Bias ± SD | LOA | Bias ± SD | LOA | Bias ± SD | LOA |
| EDV [ml] | 0.46 ± 1.24 | -1.98 - 2.87 | 0.97 ± 1.09 | -1.17 - 3.11 | 0.81 ± 1.53 | -2.19 - 3.8 |
| ESV [ml] | 0.46 ± 0.69 | -0.9 - 1.82 | 0.97 ± 1.11 | - 1.21 - 3.14 | 0.3 ± 1.83 | -3.28 - 3.89 |
| SV [ml] | 0.079 ± 0.40 | -0.71 - 0.87 | -0.04 ± 0.31 | -0.65 - 0.57 | 0.04 ± 0.2 | -0.35 - 0.43 |
| EF [%] | -1.06 ± 3.38 | -7.68 - 5.57 | -2.15 ± 2.67 | -7.34 - 3.07 | -2.18 ± 4.26 | -10.53 - 6.17 |
| dp/dt_max_ [mmHg/s] | -149.6 ± 297.8 | -698 - 398.9 | 27 ± 223 | -411 - 465 | -62 ± 88 | -234 - 111 |
| dp/dt_min_ [mmHg/s] | -68.97 ± 290.2 | -637.8 - 499.8 | 266 ± 309 | -338 - 871 | 38 ± 161 | -278 - 354 |
| P_max_ [mmHg] | 2.61 ± 4.39 | -5.99 - 11.22 | 3.25 ± 6.0 | -8.5 - 15 | 1.97 ± 6.48 | -10.72 - 14.66 |
| EDP [mmHg] | 0.53 ± 1.24 | -1.91 - 2.96 | 0.65 ± 2.58 | -4.41 - 5.71 | 1.21± 1.89 | -2.5 - 4.92 |
| Heart rate [/min] | -1.03 ± 15.02 | -30.47 - 28.42 | 5.48 ± 6.42 | -7.1 - 18.05 | 4.7 ± 8.6 | -12.18 - 21.58 |

SD, standard deviation; LOA, limits of agreement; EDV, enddiastolic volume; ESV, endsystolic volume; SV, stroke volume; EF, ejection fraction; dp/dt_max_ and _min_, maximal and minimal rate of pressure change over time; P_max_, maximal pressure; EDP, enddiastolic pres
