# Supplementary material for: 3D Real-Time Echocardiography Combined with Mini Pressure Wire Generate Reliable Pressure-Volume Loops in Small Hearts
Source: PLoS One. 2016 Oct 24;11(10):e0165397. doi: 10.1371/journal.pone.0165397 (PMC5077139; doi:10.1371/journal.pone.0165397)
Supplement: S2 Table — (DOC) [file pone.0165397.s002.doc]

| **S2 Table. Comparison of parameters obtained by 3DE and conductance technology under different hemodynamic conditions (Student t-test; mean ± SD)** | | | | | | |
| --- | --- | --- | --- | --- | --- | --- |
|  | **PVR_3D_** | | | **PVR_Cond_** | | |
|  | baseline | phenylephrine | esmolol | baseline | phenylephrine | esmolol |
| EDV [ml] | 15.31 ± 3.48 | 15.64 ± 3.80 | 16.4 ± 3.94 | 14.86 ± 3.61 | 14.66 ± 3.29 | 15.59 ± 4.09 |
| ESV [ml] | 6.75 ± 1.95 | 8.47 ± 2.83* | 9.57 ± 2.94*† | 6.28 ± 1.73 | 7.5 ± 2.3* | 9.27 ± 3.43*† |
| SV [ml] | 8.68 ± 1.83 | 7.33 ± 2.1* | 7.12 ± 1.89* | 8.61 ± 1.76 | 7.37 ± 2.17* | 7.08 ± 1.79* |
| EF [%] | 56.23 ± 4.07 | 46.5 ± 9.26* | 46.24 ± 6.51* | 57.28 ± 5.59 | 48.65 ± 8.26* | 48.42 ± 5.76* |
| dp/dt_max_ [mmHg/s] | 2310 ± 457 | 3383 ± 865* | 1796 ± 490*† | 2460 ± 467 | 3356 ± 868* | 1858 ± 496*† |
| dp/dt_min_ [mmHg/s] | -2299 ± 508 | -2574 ± 731 | -1922 ± 417† | -2230 ± 437 | -2841 ± 921 | -1960 ± 413† |
| P_max_ [mmHg] | 96.7 ± 15.3 | 149.9 ± 22.6* | 108 ± 14.9† | 94.8 ± 13.3 | 141.8 ± 25.1* | 102 ± 17.7† |
| EDP [mmHg] | 10.66 ± 4.1 | 16.65 ± 8.31 | 17.33 ± 5.49* | 10.8 ± 3.96 | 15.99 ± 6.56* | 16.12 ± 4.23* |
| ESP [mmHg] | 94.86 ± 15.21 | 141.3 ± 25.86* | 104.2 ± 19.92† | 92.09 ± 16.48 | 136.7 ± 27.78* | 97.3 ± 13.82† |
| Ees [mmHg/ml] | 19.18 ± 6.0 | 28.25 ± 9.59* | 18.44 ± 6.62† | 19.14 ± 5.63 | 26.16 ± 7.63* | 16.79 ± 5.92† |
| Ea [mmHg/ml] | 11.36 ± 2.52 | 20.73 ± 6.21* | 16.85 ± 5.67*† | 11.57 ± 4.01 | 19.11 ± 5.63* | 16.36 ± 3.99*† |
| Ees/Ea | 1.63 ± 0.38 | 1.46 ± 0.68 | 1.11 ± 0.43* | 1.62 ± 0.41 | 1.45 ± 0.64 | 1.01 ± 0.32* |
| tau [s] | 19.25 ± 3.46 | 24.45 ± 9.18 | 26.67 ± 9.23*† | 18.56 ± 3.57 | 23.95 ± 9.28 | 26.16 ± 9.86* |
| EDV_10_ [ml] | 14.34 ± 4.21 | 14.68 ± 3.0 | 15.37 ± 4.27 | 13.62 ± 4.01 | 14.64 ± 3.24 | 14.93 ± 4.65 |
| Heart rate [/min] | 124 ± 22 | 129 ± 25 | 128 ± 17 | 125 ± 22 | 123 ± 24 | 124 ± 17 |

* significant compared to baseline (p<0.05), † significant compared to phenylephrine (p<0.05)

SD, standard deviation; EDV, enddiastolic volume; ESV, endsystolic volume; SV, stroke volume; EF, ejection fraction; dp/dtmax and min, maximal and minimal rate of pressure change over time; Pmax, maximal pressure; EDP, enddiastolic pressure; ESP, endsystolic pressure; Ees, endsystolic elastance; Ea, arterial elastance; Ees/Ea, ventriculoarterial coupling; EDV10, indexed enddiastolic volume at an enddiastolic pressure of 10 mmHg
