# Supplementary material for: 3D Real-Time Echocardiography Combined with Mini Pressure Wire Generate Reliable Pressure-Volume Loops in Small Hearts
Source: PLoS One. 2016 Oct 24;11(10):e0165397. doi: 10.1371/journal.pone.0165397 (PMC5077139; doi:10.1371/journal.pone.0165397)
Supplement: S3 Table — (DOCX) [file pone.0165397.s003.docx]

| **S3 Table. Comparison of pressure-volume relations obtained by 3DE (PVR_3d_) between open vs. closed chest measurements at baseline conditions** | | | | |
| --- | --- | --- | --- | --- |
|  | **Open chest** | **Closed chest** | **Bland-Altman (Open-Closed)** | |
|  | Mean ± SD | Mean ± SD | Bias ± SD | LOA |
| EDV [ml] | 15.67 ± 3.83 | 14.93 ± 3.17 | 2.01 ± 2.83 | -3.55 - 7.56 |
| ESV [ml] | 6.91 ± 2.2 | 5.97 ± 2.42 | 1.58 ± 2.32 | -2.97 - 6.13 |
| SV [ml] | 8.74 ± 1.84 | 8.96 ± 1.43 | 0.39 ± 1.46 | -2.47 - 3.24 |
| EF [%] | 56.39 ± 4.75 | 61.08 ± 8.71 | -5.01 ± 8.98 | -22.61 - 12.6 |
| EDV_10_ [ml] | 14.74 ± 4.03 | 14.1 ± 3.0 | 1.88 ± 2.96 | -3.93 - 7.68 |
| EDP mmHg] | 11.5 ± 4.25 | 12.61 ± 4.66 | -0.8 ± 2.87 | -6.42 - 4.81 |
| P_max_ [mmHg] | 98.91 ± 14.86 | 98.96 ± 12.54 | 0.66 ± 8.38 | -15.76 - 17.09 |
| dp/dt_max_ [mmHg/s] | 2618 ± 932.7 | 2388 ± 844.1 | -101.1 ± 284.8 | -659.2 - 457.1 |
| dp/dt_min_ [mmHg/s] | -2342 ± 569.2 | -2273 ± 461.6 | -36.81 ± 533.7 | -1083 - 4009 |
| tau [s] | 17.97 ± 4.77 | 19.37 ± 7.38 | 0.18 ± 5.54 | -10.68 - 11.04 |
| Ees [mmHg/ml] | 18.77 ± 6.92 | 17.39 ± 7.6 | 1.34 ± 8.89 | -16.09 - 18.77 |
| Ea [mmHg/ml] | 11.45 ± 2.89 | 10.75 ± 1.7 | 0.15 ± 2.25 | -4.55 - 4.23 |
| ESP [mmHg] | 94,91 ± 14.7 | 94,24 ± 13.6 | 1.6 ± 8.9 | -15.9 – 19.1 |
| Heart rate[/min] | 133.3 ± 24.98 | 130.5 ± 15.75 | -8.8 ± 14.32 | -36.88 - 19.27 |

*= significant difference between closed and open chest condition (p<0.05)

SD, standard deviation; LOA, limits of agreement; EDV, enddiastolic volume; ESV, endsystolic volume; SV, stroke volume; EF, ejection fraction; EDV_10_, indexed enddiastolic volume at an enddiastolic pressure of 10 mmHg; EDP, enddiastolic pressure; P_max_, maximal pressure; dp/dt_max_ and _min_, maximal and minimal rate of pressure change over time; Ees, endsystolic elastance; Ea, arterial elastance; Ees/Ea, ventriculoarterial coupling
